# Long noncoding RNAs in cancer: mechanisms of action and technological advancements

**DOI:** 10.1186/s12943-016-0530-6

**Published:** 2016-05-27

**Authors:** Nenad Bartonicek, Jesper L. V. Maag, Marcel E. Dinger

**Affiliations:** Genome Informatics, Genomics & Epigenetics Division, Garvan Institute of Medical Research, Sydney, NSW Australia; Faculty of Medicine, St Vincent’s Clinical School, University of New South Wales, Sydney, NSW Australia

## Abstract

The previous decade has seen long non-coding RNAs (lncRNAs) rise from obscurity to being defined as a category of genetic elements, leaving its mark on the field of cancer biology. With the current number of curated lncRNAs increasing by 10,000 in the last five years, the field is moving from annotation of lncRNA expression in various tumours to understanding their importance in the key cancer signalling networks and characteristic behaviours. Here, we summarize the previously identified as well as recently discovered mechanisms of lncRNA function and their roles in the hallmarks of cancer. Furthermore, we identify novel technologies for investigation of lncRNA properties and their function in carcinogenesis, which will be important for their translation to the clinic as novel biomarkers and therapeutic targets.

**Electronic supplementary material:**

The online version of this article (doi:10.1186/s12943-016-0530-6) contains supplementary material, which is available to authorized users.

## Background

Our understanding of cancer biology was drastically changed by the genomic revolution of the last decade, marked by the conclusion of the human genome project and the development of novel DNA sequencing technologies [1, 2]. The complete human genome sequence provided a framework for comparison of populations with cancer susceptibility, allowing for clinical prognosis based on mutations in genes such as BRCA1/2 or differential treatment based modifications in KRAS and BRAF [3–5]. Sequencing of individual tumours revealed the prevalence of acquired DNA damage compared to the germline mutations, which allowed identification of footprints for individual mutagens and gave us important insights into tumour heterogeneity and evolution [6–10]. Parallel to the progress in genomics, advances in transcriptomics initiated functional annotation of numerous genomic loci associated with cancer that do not overlap protein-coding genes – the noncoding genome.

Large-scale cDNA sequencing projects, together with technological advancements such as tiling arrays and the next generation RNA sequencing provided an unprecedented view of the transcriptome complexity [11–15]. Surprisingly, only 1–2 % of the whole genome encodes proteins, with evidence of at least 80 % of the remainder being actively transcribed [11, 16]. These non-coding portions of the genome produce a large variety of mostly regulatory RNAs that differ in their biogenesis, properties and function, and are separated by their size into short, such as miRNAs (reviewed in [17]) and long (>200 nt) RNAs [12, 18–20]. The heterogeneous category of long non-coding RNAs (lncRNA) are especially abundant, accounting for 16,000 curated records in the current Gencode annotation (v.23) [21] with for all lncRNA loci in the human genome numbering as high as 60,000 [22].

lncRNAs remained elusive even in the genomics era due to their low expression levels and their presence in specific cell types, tissues or narrow time frames [23–25]. They were identified as a class of RNA molecules in 2002 [26], even though some lncRNA such as H19 and Xist were known since the early 1990s [27, 28] Analogous to protein coding genes but with low coding potential, these RNAs are usually transcribed by RNA polymerase II (Pol II), spliced, and mostly polyadenylated [12, 13]. Similarly, lncRNA promoters are enriched for active histone modifications typical of Pol II occupancy: H3K4me3, H3K9ac and H3K27ac [20, 29]. Even though the sequence of lncRNAs evolves rapidly, especially compared to their 3D structure, their tissue specificity as well as promotor sequences remain conserved as protein-coding genes [30–32]. The heterogeneity of lncRNAs resonates in the diversity of their functions; lncRNAs interact with DNA, proteins and other RNAs to participate in processes from transcription, intracellular trafficking to chromosome remodelling as reviewed previously [29, 33]).

lncRNAs have been observed to regulate complex cellular behaviours such as growth, differentiation and establishment of cell identity that are commonly deregulated in cancer [34–36]. Some have already been linked to poor prognosis in multiple tumour types and have a clinical relevance as biomarkers. In this review we will focus on the molecular mechanisms of function for cancer-associated lncRNAs, their involvement in cancer hallmarks and provide information on the most recent advances in technologies for their identification and functional interrogation.

### Identification of lncRNAs in cancer

**Fig. 1:**
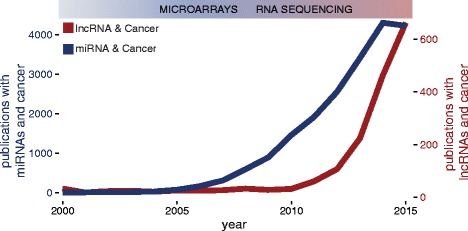
Interest for lncRNAs (*red*) in the cancer scientific community compared to miRNAs (*blue*). The y-axis represents the number of publications and the x-axis represents time. Data was obtained by searching Pubmed for ‘lncRNA cancer’ or ‘miRNA cancer’. Data from 2016 was not used in the graph. Publications with terms ‘miRNA’ and ‘cancer’ plateau in 2015

**Fig. 2:**
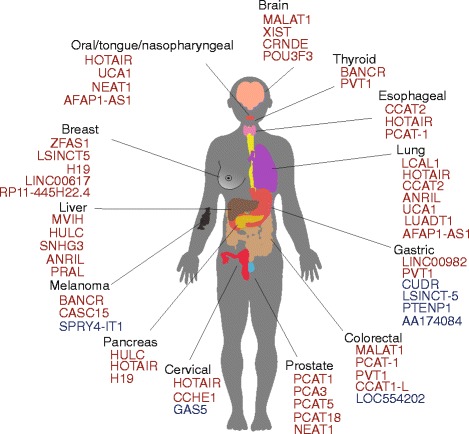
Some lncRNAs associated with cancers. The colour represents either upregulated (*red*) or downregulated (*blue*) compared to normal tissues

lncRNAs were initially observed in carcinogenesis due to their differential expression compared to normal tissues. High expression in tumour tissues of some of the first identified lncRNAs such as h19, MALAT1 and PCA3 was recognised before the availability of next-generation sequencing technologies [37–39]. RNA sequencing allowed a large-scale assessment of differential expression of lncRNAs comparing cancer to normal tissues, with a large number of lncRNAs showing aberrant expression, similar to the influence microarrays had on the miRNA field (Fig. 1). Recently, a number of lncRNAs have been systematically identified in numerous cancer transcriptomes, either by overlap of sequencing libraries with previously annotated GENCODE lncRNAs, or by *de novo* assembly of all available public datasets [22, 40], marking their presence in the majority of cancer types (Fig. 2, Additional file 1: Table S1). Novel RNA-seq techniques such as CaptureSeq, that enriches transcript libraries for specific oligonucleotides designed for genomic regions of interest, will improve observation of rare or lowly abundant transcripts from gene deserts associated with cancer [41].

There are two main drivers for altered expression of lncRNAs in tumours. First, abundance of some lncRNAs can be altered due to the cancer-induced change of copy number of their genomic loci. This phenomenon has been observed for FAL1 in ovarian and PRAL in hepatocellular carcinoma [42, 43]. Second, expression of some cancer-associated lncRNAs can be initiated by oncogenes acting as transcription factors. Two crucial genes implicated in multiple tumour types, Myc and p53, act as transcription factors for a large number of lncRNAs [44–46]. Some of these lncRNAs modulate activity of their respective TFs in a feedback loop, for example MINCR with Myc and MEG3 for p53, which makes them potential candidates for therapeutic targeting [47, 48].

Presence of lncRNAs in specific tumours can also be observed based on their overlap with the cancer risk loci identified through genome-wide association studies (GWAS). For example, association of ANRIL with glioma and basal cell carcinoma as well as PTCSC3 with thyroid cancer were discovered based on the known risk loci established through genotyping of cancer patients [49, 50] Recently, CASC15 and NBAT1 were identified through GWAS of neuroblastoma, a cancer that mostly affects children and has a poorly explained genetic background. Both CASC15 and NBAT1 are part of the 6p22 locus that contains SNP rs6939340, associated with metastatic disease and poor event free survival [51, 52]. CASC15 acts as a tumour suppressor and is associated with advanced tumour stages and poor patient survival, while NBAT1 seems to negatively regulate transcription factor NRSF (neuron restrictive silencing factor) [52, 53]. Furthermore, CARLo-5 was identified as significantly correlated with the rs6983267 allele, a single nucleotide polymorphism (SNP) in the region for Myc enhancers. This lncRNA is associated with increased cancer susceptibility and has a function in cell-cycle regulation and tumour development [54]. Since more than 80 % of disease associated SNPs fall into intronic and intergenic regions, it is likely that future development of more sensitive technologies for RNA detection will be essential in defining novel cancer-associated lncRNAs [55].

### Mechanisms of lncRNA function in carcinogenesis

Although lncRNAs are scarcely functionally annotated [56], their mechanism of action can be separated based on their influence on chromatin state and methylation, stability of proteins and complexes or by acting as a sponge for miRNA inhibition (reviewed in [29]).

#### Chromatin state and methylation

Chromatin remodelling was one of the first identified functions of lncRNAs. Epigenetic remodelling is frequently achieved through interaction of a lncRNAs with PRC2 (reviewed in [57]), a protein from the polycomb complex that introduces chromatin inactivation by establishing inhibitory H3K427me3 histone marks [58]. These lncRNAs are sometimes expressed antisense to the target gene involved in cancer, such as in the case of ANRIL with CDKNA/B and asFGFR2 with FGFR2 [59, 60], suggesting a possibility for a *cis*-acting activity. PCE3, as the first lncRNA involved in cancer was only recently found to have the same mode of action, with the discovery of its antisense protein oncogene PRUNE2 [61]. However, it has also been observed that PRC2 lacks specificity in binding RNAs that recruit it, providing a potential explanation why such a large number of lncRNAs influence chromatin remodelling [62]. Besides acting through PRC2, some lncRNAs such as Kcnq1ot1, TARID, AS1DHRS4 and DACOR1 recruit DNA methyltransferases directly to modify chromatin conformation, or they modify nucleosome positioning through SWI/SNF complex as in the case of SChLAP1 [63–67]. Other lncRNAs, like Firre, bind chromatin remodelers cohesin and CTCF to change chromatin state of the whole chromosomes in the process of X chromosome inactivation [68]. lncRNAs can also act as chromatin activators, as in the case of HOTTIP and CCAT1-L that regulate chromosome looping in their proximity to deposit activating H3K4me3 mark on gene promoters [69–71].

#### Stability of proteins or protein complexes

A large number of lncRNAs exert their oncogenic function through direct interaction with proteins or protein complexes as scaffolds or allosteric activators/inhibitors. CTBP1-AS, CCTA2 and ZBTB7A interact with transcription factors and modify their activity [72–74]. Some lncRNAs can be used as a scaffold for assembly of whole protein complexes, for example HOTAIR for the HBXIP/Hotair/LSD1 complex [75], NEAT1 for paraspeckle proteins [76, 77], BCAR4 for binding of SNIP1 and PNUTS [78], PRAL for HSP90 and p53 [43]. Furthermore, TERRA recruits a complex of chromatin modifiers to regulate telomere maintenance in response to a variety of cellular signals [79]. Finally, some lncRNAs such as GAS5 bind to nuclear receptors, in this case the glucocorticoid receptor [80].

#### Competing endogenous RNAs

Some lncRNAs have recently been found to act as competing endogenous RNAs (ceRNAs), by binding miRNAs (“sponging”) and reducing their inhibitory effect on their natural targets (reviewed in [81]). There are numerous examples of lncRNA sponges involved in cancer progression. In lung cancer, UCA1 up-regulates a potent oncogene ERBB4 by binding miR-193-3p [82]. In gastric cancer, MEG3 upregulates Bcl-2 by sequestering miR-181-a [83]. Similarly, ZFAS1 binds miR-150 in hepatocellular carcinoma, Linc-RoR binds miR-145 in endometrial cancer stem cells and CASC2 regulates concentration of miR-21 [84–86].

In addition to the previously described mechanisms, lncRNAs have recently been observed to employ the following set of diverse strategies. lncRNAs can modify the phosphorylation state of proteins by masking phosphorylation motifs, like LINK-a and HIF1a [87]. Furthermore, some lncRNAs such as NORAD act as sponges for a whole set of proteins, in this case the PUMILIO family that would otherwise drive chromosomal instability by repressing mitotic, DNA repair, and DNA replication factors [88]. Interestingly, some lncRNAs form DNA-RNA triplexes that regulate expression of oncogenes either in *cis* for Khps1, or in *trans* for MEG3 [89, 90]. Other lncRNAs such as Uc.283 + A control production of miRNAs by influencing processing of the pri-miRNA transcripts, in this case pri-miR-195 [91].

In summary, lncRNAs act through an increasingly wide range of mechanisms that compete with proteins in terms of their diversity and regulatory potential. In the next section we discuss how these mechanisms impact upon cell transformation to cancer phenotype.

### Hallmarks of cancer

In 2000, Hanahan and Weinberg described six properties required for cell transformation, termed hallmarks of cancer. These included self-sustained growth signalling, insensitivity to growth inhibition, apoptosis avoidance, uncontrolled proliferation, angiogenesis and metastasis [92, 93]. lncRNAs as regulatory molecules have been implicated in the majority of these functions (reviewed in [94]), and key patterns are starting to emerge.

#### Self-sustained growth signalling

lncRNAs promote self-sufficiency in growth signals mostly by acting on the activation on the first step of the signal transduction, the signal receptors. Multiple lncRNAs have been observed to specifically bind nuclear receptors either alone or in a ribonucleoprotein complexes (reviewed in [95]). SRA1 serves as a scaffold to stabilize the estrogen receptor [96, 97], while GAS5 acts as the competitive inhibitor of the glucocorticoid receptor [80]. Instead of activating the signal receptors, some lncRNAs such as PVT1 affect the proliferation by regulating the receptor abundance, as is demonstrated for PVT1 and thyroid-stimulating hormone [98].

#### Insensitivity to growth inhibition

Growth inhibition or its evasion can also be regulated by lncRNAs, and mostly involves influence on tumour suppressors that regulate cell cycle such as cyclins, CDKs, CDK inhibitors and p53 (reviewed in [99]). This can be achieved by chromatin repression through PRC complex, as detailed in the previous section. Using this mechanism PANDA represses protein CDKN1A through PRC1 while ANRIL repress their targets tumour suppressor p15 (CDKN2B) through PRC2 [59, 100, 101]. Some lncRNAs regulate expression of tumour suppressors by influencing various parts of transcription and translation. Initiation of transcription can be influenced by scaffolding of transcription factor complexes, as in the case of LincRNA-p21 and p21 (inhibitor of CDK2) [102]. Transcription elongation can be modified by destabilization of mRNA transcripts, as exemplified by gadd7 and Cdk6 [103]. Finally, transcript stability and translation can be regulated post-transcriptionally by diminishing the role of miRNAs, as in the case of PTENP1 acting as a competitive endogenous RNA to inhibit miRNA repression of PTEN [104]. For some of the lncRNAs like CASC15-S, the direct mechanism of growth inhibition is unknown, but the lack of its expression increases cancer growth and migratory capacity [53].

#### Avoiding apoptosis

Apoptosis or controlled cell death is one of the key pathways for control of carcinogenesis (reviewed in [36]). Some lncRNAs act on regulation of transcription of key apoptotic genes. For example, lncRNA INXS is expressed from the intron of BCL-X and regulates its splicing into a pro-apoptotic isoform BCL-XS [105]. A recently discovered lncRNA PRAL induces apoptosis by stabilizing a complex between HSP90 and p53 [43]. Several other lncRNAs have been implicated in apoptosis such as SPRY4-IT1 [106], HOXA-AS2 [107] and uc002mbe.2 [108], but the details of the mechanism of action remain still unknown.

#### Uncontrolled proliferation

Maintenance of telomeres as nucleoprotein structures that stabilize ends of chromosomes is a key requirement for limitless replicative potential of cancer cells. Telomeres shorten in dividing cells, so a ribonucleoprotein complex telomerase is required to elongate telomeric repeats through reverse transcription of an internal template RNA. Shortening of telomeres induces production of a lncRNAs TERRA, that is transcribed from the subtelomeric regions [109]. Under normal conditions, TERRA represses its expression through chromatin modifications, but when activated can recruits protein complexes for homology-directed repair of shortened or damaged telomeric sequences [79].

#### Promotion of angiogenesis

Multiple lncRNAs have been implicated in regulation of nutrient supply to tumours, mostly by regulating vascular endothelial growth factor (VEGF) that is essential for formation of blood vesicles. Transcription of VEGF was recently reported to be regulated by lncRNAs HOTAIR [110] and MIAT [111]. MIAT sequesters mir-150-5p that is required for repression of VEGF, resulting in microvascular dysfunction and decreased metastasis after MIAT knockdown. MVIH also influences production of VEGF, though indirectly through phosphoglycerate kinase 1 (PGK1) [112]. Finally, MALAT1 has been observed to promotes angiogenic sprouting and migration when expressed in endothelial cells [113].

#### Tissue invasion and metastasis

Multiple lncRNAs increase invasiveness of cancer cells and facilitate metastasis. Examples of these include h19 [114], MALAT1 in colorectal and nasopharyngeal carcinoma [115], SPRY4-IT1 in melanoma [106], HOTAIR [116], AFAP1-AS1 [117], and CCAT2 [118] in lung cancer, lincRNA-RoR in breast cancer [119], LEIGC in gastric cancer [120] and lncRNA-ATB in hepatocellular carcinoma [121]. Out of these, only lincRNA-RoR and lncRNAs-ATB have a suggested mechanism of action in tissue invasion. lincRNA-RoR likely serves as a “sponge” for miR-145 that is important for regulation of ADP-ribosylation factor 6, a protein involved in invasion of breast cancer cells [119]. Similarly, lncRNA-ATB, acts as a ceRNA to reduce the effect of the miR-200 family targets ZEB1 and ZEB2, two transcription factors that promote cell motility and metastasis [121].

lncRNAs can be involved in a number of other processes related to cancer. Some lncRNAs promote a metabolic switch to glycolysis and lactic acid fermentation termed the Warburg effect [122]. lincRNA-p21 regulates the Warburg effect by preventing ubiquitination of hypoxia-inducible factor-1 (HIF-1), a key transcription factor that promotes upregulation of glycolysis and downregulation of oxidative phosphorylation [123]. Several lncRNAs have been observed as essential for DNA repair by homologous recombination (HR): ANRIL, PCAT1 and DDSR1. Although the mechanism of ANRIL in HR remains unknown, PCAT1 posttranslationally inhibits BRCA2 [124], while DDSR1 is suggested to interact with BRCA1 [125]. Finally, there are implications of lncRNAs on cancer therapies through expression of drug exporters. For example, MRUL promotes expression of ABCB1 that is essential for multidrug-resistance in gastric cancer cell lines [126].

##### Novel techniques for lncRNA interrogation

The number of annotated transcribed genomic elements has increased by 100 % in the last decade, the majority of which are in the non-coding space and have a defined function in less than 1 % of cases [56]. Such a vast number of novel genetic players presents a great potential for clinical applications, especially in view of cancer as a genomic disease. However, it also requires a thorough rethinking of our basic premises on biological systems, pathway structure and information transfer, as well as a clear technological strategy to identification of their function.

The first challenge is presented by the lack of an exhaustive definition of the full cancer transcriptome, regardless of the cell or tissue type. Currently, a major obstacle to analysis of cancer transcriptome is the alignment of sequence reads to the consensus human genome. Ideally, all the reads would be aligned to a genome sequenced by single-molecule DNA sequencing, but the cost and the quality of this technology are still keeping it away from mainstream research. The next issue is the limited dynamic range of transcript detection for RNA sequencing. This can already be solved by applying the recently developed CaptureSeq method for targeted enrichment of transcripts from specific regions of interest [41]. Furthermore, long read sequencing will be essential for discovery of lncRNAs isoforms and novel exons [127]. In combination with single cell sequencing it will allow identification of individual lncRNAs species from cancer subpopulations, avoiding the heterogeneity of tissue mixture.

After defining the non-coding elements of the transcriptome, the second challenge is the systematic identification of lncRNAs properties that could lead to identifying their cellular function. This can be achieved by investigating their location in the cellular compartments, structural properties as well as possible interactors.

Quantified localization of lncRNAs through microscopy techniques can provide important information about their properties. RNA-Fish as an established technique for RNA localisation has recently been used to identify subcellular location of multiple lncRNAs, in addition to their expression across a population of cells, spatio-temporal behaviour and coexpression with proximal mRNAs [128].

Structure of biological molecules is vital to their function, and several techniques have been developed to investigate secondary and tertiary structures of lncRNAs. Techniques such as Parallel Analysis of RNA Structure (PARS) [129] and Fragmentation Sequencing (FragSeq) [130] sequence RNAs after specific cleavage of single (FragSeq) or single and double stranded (PARS) nucleic acids, allowing for identification of loops in RNA-structure. Another way to investigate structure is to tag the flexible 2′-hydroxyl groups in the RNA backbone by Selective 2′-hydroxyl Acylation and Primer Extension (SHAPE) [131]. Finally, similarly to DNA, RNA can be edited with chemical modifications that modify its structure and binding properties. Two established methods can be used to identify methylated RNA sites: Methylated RNA Immunoprecipitation with next-generation sequencing (MeRIP) [132], or its adaption for hydroxymethylcytosine sites – hMeRIP [133]. Another common RNA-modification is chemical change of nucleotides adenosin to inosin, which can be detected by inosine chemical erasing sequencing (ICE-seq) [134].

Assessing the function of lncRNAs by identifying their binding partners can be performed depending on the type of interaction. Binding of RNA to DNA or proteins can be assessed with ChIRP-seq or ChIRP-MS respectively (Chromatin Isolation by RNA purification followed by sequencing or mass spectrometry) [135, 136]. The specificity of ChIRP is guaranteed by selection of only those RNA that are bound by biotinylated oligonucleotides, similar to RAP [137] and CHART [138], as well as by crosslinking of RNA with DNA or protein by UV or formalin. A recent modification to the protocol can detect individual RNA domains that interact with DNA, RNA or proteins [139]. Instead of biotinylated oligonucleotides, RNA-guided chromatin conformation capture (R3C) reverse-transcribes RNA bound to DNA into cDNA with biotin labelling and joins it with the adjacent genomic DNA with T4 DNA ligase, allowing for streptavidin selection and sequencing [140]. Furthermore, identification of lncRNAs that bind to a protein of interest such as PRC2 [141] can be performed through RNA Immunoprecipitation [142] that was later coupled with sequencing (RIP-Seq) [65]. The specificity of RIP has been improved in by UV crosslinking of RNA and protein in Cross-Linking ImmunoPrecipitation (CLIP) [143] and the later modifications with sequencing (HITS-CLIP) [144] and iClip [145]. Finally, the affinity of a protein for multiple RNA can be assessed in a high-throughput manner. This can be achieved either on a microfluidic platform by RNA-mechanically Induced Trapping of Molecular Interactions (RNA-MITOMI) [146], or on a flow cell in RNA-MaP (massively parallel array) [147].

The final challenge in defining lncRNA functions is developing loss- and gain-of-function lncRNA studies. The RNA interference technology is being supplemented by the powerful CRISPR/Cas-9 system, a newly developed genome-editing technology that allows easier manipulation of lncRNAs behaviour [148]. CRISPR allows multiple types of manipulation, from deletion of various parts of genomic lncRNAs loci, to insertion of promoters, and novel exons. A recent modification of the CRISPR technique that was developed in Rinn group allows insertion of RNA domains to genomic loci, allowing for identification of in cis behaviour of lncRNAs [149].

## Conclusion

Long non-coding RNAs are fine-tuners and regulators of key biological processes. Though we have only started to annotate their function in various aspects of cell transformation and metastasis, they are already filling in the major gaps of our understanding of cancer biology. It will be exciting to see the next decade migrate from the perception of lncRNAs as a side act in biological regulation to the center of new biological concepts, paradigms and drug therapies. Watch this space.

## Abbreviations

ceRNA, competing endogenous RNAs; CHART, capture hybridization of analysis of RNA targets; ChIRP-seq or ChIRP-MS, chromatin isolation by RNA purification followed by sequencing or mass spectrometry; CLIP-seq, cross-linking immunoprecipitation sequencing; GWAS, genome-wide association studies; hMeRIP, hydroxymethylcytosine sites in RNA; lncRNA, long noncoding RNA; MeRIP, methylation RIP-seq; PARS, parallel analysis of RNA structure; RAP, RNA antisense purification; RIP, RNA immunoprecipitation; RNA-MaP, RNA on a massively parallel array RNA-MITOMI, RNA-mechanically induced trapping of molecular interactions; SHAPE, selective 2′-hydroxyl acylation and primer extension; SNP, single nucleotide polymorphism

## Additional file

Additional file 1: Table S1 lncRNAs and references for Fig. 2. (PDF 106 kb)
