# Supplementary material for: Long noncoding RNAs in cancer: mechanisms of action and technological advancements
Source: Mol Cancer. 2016 May 27;15:43. doi: 10.1186/s12943-016-0530-6 (PMC4884374; doi:10.1186/s12943-016-0530-6)
Supplement: Additional file 1: Table S1 — lncRNAs and references for Fig. 2. (PDF 106 kb) [file 12943_2016_530_MOESM1_ESM.pdf]

Supplementary table 1. lncRNAs and references for figure 2.

| **Organ** | **lncRNA** |
|---|---|
| Brain | MALAT1 [1] |
| | CRNDE [2] |
| | POUF3 [3] |
| Thyroid | BANCR [4] |
| | PVT1 [5] |
| Esophagus | CCAT2 [6] |
| | HOTAIR [7] |
| | PCAT-1 [8] |
| Lung | LCAL1 [9] |
| | HOTAIR [10] |
| | CCAT2 [11] |
| | ANRIL [12] |
| | UCA1 [13] |
| | LUADT1 [14] |
| | AFAP1-AS1 [15] |
| Gastric | LINC00982 [16] |
| | PVT1 [17] |
| | CUDR [18] |
| | LSINCT-5 [18] |
| | PTENP1 [18] |
| | AA174084 [19] |
| Colorectal | MALAT1 [20] |
| | PCAT-1 [21] |
| | PVT1 [22] |
| | CCAT1-L [23] |
| | LOC554202 [24] |
| Prostate | PCAT1 [25] |
| | PCA3 [26] |
| | PCAT5 [27] |
| | PCAT18 [28] |
| | NEAT1 [29] |
| Cervical | HOTAIR [30] |
| | CCHE1 [31] |
| | GAS5 [32] |
| Pancreas | HULC [33] |
| | HOTAIR [34] |
| | H19 [35] |
| Melanoma | BANCR [36] |
| | CASC15 [37] |

| **Organ** | **lncRNA** |
|---|---|
| Melanoma (continued) | SPRY4-IT1 [38] |
| Liver | MVIH [39] |
| | HULC [40] |
| | SNHG3 [41] |
| | ANRIL [42] |
| Breast | ZFAS1 [43] |
| | LSINCT5 [44] |
| | H19 [45] |
| | LINC00617 [46] |
| | RP11-445H22.4 [47] |
| Oral/Tongue/nasopharyngeal | HOTIAR [48] |
| | UCA1 [49] |
| | NEAT1 [48] |
| | AFAP1-AS1 [50] |

1. Ma K-X, Wang H-J, Li X-R, Li T, Su G, Yang P, Wu J-W: **Long noncoding RNA MALAT1 associates with the malignant status and poor prognosis in glioma.** *Tumor Biol* 2015, **36**:3355–3359.

2. Wang Y, Wang Y, Li J, Zhang Y, Yin H, Han B: **CRNDE, a long-noncoding RNA, promotes glioma cell growth and invasion through mTOR signaling**. *CANCER LETTERS* 2015, **367**:122–128.

3. Guo H, Wu L, Yang Q, Ye M, Zhu X: **Functional linc-POU3F3 is overexpressed and contributes to tumorigenesis in glioma**. *Gene* 2015, **554**:114–119.

4. Wang Y, Guo Q, Zhao Y, Chen J: **BRAF-activated long non-coding RNA contributes to cell proliferation and activates autophagy in papillary thyroid carcinoma**. *Oncology …* 2014.

5. Zhou Q, Chen J, Feng J, Wang J: **Long noncoding RNA PVT1 modulates thyroid cancer cell proliferation by recruiting EZH2 and regulating thyroid-stimulating hormone receptor (TSHR)**. *Tumor Biol* 2015.

6. Zhang X, Xu Y, He C, Guo X, Zhang J, He C, Zhang L, Kong M, Chen B, Zhu C: **Elevated expression of CCAT2 is associated with poor prognosis in esophageal squamous cell carcinoma.** *J Surg Oncol* 2015, **111**:834–839.

7. Chen F-J, Sun M, Li S-Q, Wu Q-Q, Ji L, Liu Z-L, Zhou G-Z, Cao G, Jin L, Xie H-W, Wang C-M, Lv J, De W, Wu M, Cao X-F: **Upregulation of the long non-coding RNA HOTAIR promotes esophageal squamous cell carcinoma metastasis and poor prognosis.** *Mol Carcinog* 2013, **52**:908–915.

8. Shi W-H, Wu Q-Q, Li S-Q, Yang T-X, Liu Z-H, Tong Y-S, Tuo L, Wang S, Cao X-F: **Upregulation of the long noncoding RNA PCAT-1 correlates with advanced clinical stage and poor prognosis in esophageal squamous carcinoma**. *Tumor Biol* 2015, **36**:2501–2507.

9. White NM, Cabanski CR, Silva-Fisher JM, Dang HX, Govindan R, Maher CA: **Transcriptome sequencing reveals altered long intergenic non-coding RNAs in lung cancer**. *Genome Biology* 2014, **15**.

10. Liu X-H, Liu Z-L, Sun M, Liu J, Wang Z-X, De W: **The long non-coding RNA HOTAIR indicates a poor prognosis and promotes metastasis in non-small cell lung cancer**. *BMC Cancer* 2013, **13**:464.

11. Qiu M, Xu Y, Yang X, Wang J, Hu J, Xu L, Yin R: **CCAT2 is a lung adenocarcinoma-specific long non-coding RNA and promotes invasion of non-small cell lung cancer**. *Tumor Biol* 2014, **35**:5375–5380.

12. Naemura M, Murasaki C, Inoue Y, Okamoto H, Kotake Y: **Long Noncoding RNA ANRIL Regulates Proliferation of Non-small Cell Lung Cancer and Cervical Cancer Cells**. *Anticancer Res* 2015, **35**:5377–5382.

13. Wang H-M, Lu J-H, Chen W-Y, Gu A-Q: **Upregulated lncRNA-UCA1 contributes to progression of lung cancer and is closely related to clinical**

**diagnosis as a predictive biomarker in plasma**. *Int J Clin Exp Med* 2015, **8**:11824–11830.

14. Qiu M, Xu Y, Wang J, Zhang E, Sun M, Zheng Y, Li M, Xia W, Feng D, Yin R, Xu L: **A novel lncRNA, LUADT1, promotes lung adenocarcinoma proliferation via the epigenetic suppression of p27**. *Cell Death & Disease* 2015, **6**:e1858–.

15. Zeng Z, Bo H, Gong Z, Lian Y, Li X, Li X, Zhang W: **AFAP1-AS1, a long noncoding RNA upregulated in lung cancer and promotes invasion and metastasis**. *Tumor Biol* 2015.

16. Fei Z-H, Yu X-J, Zhou M, Su H-F, Zheng Z, Xie C-Y: **Upregulated expression of long non-coding RNA LINC00982 regulates cell proliferation and its clinical relevance in patients with gastric cancer**. *Tumor Biol* 2015.

17. Kong R, Zhang E-B, Yin D-D, You L-H, Xu T-P, Chen W-M, Xia R, Wan L, Sun M, Wang Z-X, De W, Zhang Z-H: **Long noncoding RNA PVT1 indicates a poor prognosis of gastric cancer and promotes cell proliferation through epigenetically regulating p15 and p16**. *Molecular Cancer* 2015, **14**.

18. Dong L, Qi P, Xu M-D, Ni S-J, Huang D, Xu Q-H, Weng W-W, Tan C, Sheng W-Q, Zhou X-Y, Du X: **Circulating CUDR, LSINCT-5 and PTENP1 long noncoding RNAs in sera distinguish patients with gastric cancer from healthy controls**. *Int J Cancer* 2015, **137**:1128–1135.

19. Shao Y, Ye M, Jiang X, Sun W, Ding X, Liu Z, Ye G: **Gastric juice long noncoding RNA used as a tumor marker for screening gastric cancer**. *Cancer* 2014.

20. Zheng H-T, Shi D-B, Wang Y-W, Li X-X, Xu Y, Tripathi P, Gu W-L, Cai G-X, Cai S-J: **High expression of lncRNA MALAT1 suggests a biomarker of poor prognosis in colorectal cancer**. *International Journal of Clinical and Experimental Pathology* 2014, **7**:3174.

21. Ge X, Chen Y, Liao X, Liu D, Li F, Ruan H, Jia W: **Overexpression of long noncoding RNA PCAT-1 is a novel biomarker of poor prognosis in patients with colorectal cancer**. *Med Oncol* 2013, **30**:588–6.

22. Takahashi Y, Sawada G, Kurashige J, Uchi R, Matsumura T, Ueo H, Takano Y, Eguchi H, Sudo T, Sugimachi K, Yamamoto H, Doki Y, Mori M, Mimori K: **Amplification of PVT-1 is involved in poor prognosis via apoptosis inhibition in colorectal cancers**. *Br J Cancer* 2013, **110**:164–171.

23. Xiang J-F, Yin Q-F, Chen T, Zhang Y, Zhang X-O, Wu Z, Zhang S, Wang H-B, Ge J, Lu X, Yang L, Chen L-L: **Human colorectal cancer-specific CCAT1-L lncRNA regulates long-range chromatin interactions at the MYC locus**. 2014, **24**:513–531.

24. Ding J, Lu B, Wang J, Wang J, Shi Y, Lian Y, Zhu Y, Wang J, Fan Y, Wang Z, De W, Wang K: **Long non-coding RNA Loc554202 induces apoptosis in colorectal cancer cells via the caspase cleavage cascades.** *J Exp Clin Cancer Res* 2015, **34**:100.

25. Prensner JR, Iyer MK, Balbin OA, Dhanasekaran SM, Cao Q, Brenner JC, Laxman B, Asangani IA, Grasso CS, Kominsky HD, Cao X, Jing X, Wang X, Siddiqui J, Wei JT, Robinson D, Iyer HK, Palanisamy N, Maher CA, Chinnaiyan AM: **Transcriptome sequencing across a prostate cancer cohort identifies PCAT-1, an unannotated lincRNA implicated in disease progression**. *Nat Biotechnol* 2011, **29**:742–749.

26. Salameh A, Lee AK, Cardo-Vila M, Nunes DN, Efstathiou E, Staquicini FI, Dobroff AS, Marchio S, Navone NM, Hosoya H, Lauer RC, Wen S, Salmeron CC, Hoang A, Newsham I, Lima LA, Carraro DM, Oliviero S, Kolonin MG, Sidman RL, Do K-A, Troncoso P, Logothetis CJ, Brentani RR, Calin GA, Cavenee WK, Dias-Neto E, Pasqualini R, Arap W: **PRUNE2 is a human prostate cancer suppressor regulated by the intronic long noncoding RNA PCA3**. *Proc Natl Acad Sci USA* 2015, **112**:8403–8408.

27. Ylipaa A, Kivinummi K, Kohvakka A, Annala M, Latonen L, Scaravilli M, Kartasalo K, Leppanen SP, Karakurt S, Seppala J, Yli-Harja O, Tammela TLJ, Zhang W, Visakorpi T, Nykter M: **Transcriptome Sequencing Reveals PCAT5 as a Novel ERG-Regulated Long Noncoding RNA in Prostate Cancer**. *Cancer Research* 2015, **75**:4026–4031.

28. Crea F, Watahiki A, Quagliata L, Xue H, Pikor L, Parolia A, Wang Y, Lin D, Lam WL, Farrar WL, Isogai T, Morant R, Castori-Eppenberger S, Chi KN, Wang Y, Helgason CD: **Identification of a long non-coding RNA as a novel biomarker and potential therapeutic target for metastatic prostate cancer**. *Oncotarget* 2014, **5**:764–774.

29. Chakravarty D, Sboner A, Nair SS, Giannopoulou E, Li R, Hennig S, Mosquera JM, Pauwels J, Park K, Kossai M, MacDonald TY, Fontugne J, Erho N, Vergara IA, Ghadessi M, Davicioni E, Jenkins RB, Palanisamy N, Chen Z, Nakagawa S, Hirose T, Bander NH, Beltran H, Fox AH, Elemento O, Rubin MA: **The oestrogen receptor alpha-regulated lncRNA NEAT1 is a critical modulator of prostate cancer**. *Nature Communications* 2014, **5**:5383–.

30. Li J, Wang Y, Yu J, Dong R, Qiu H: **A high level of circulating HOTAIR is associated with progression and poor prognosis of cervical cancer.** *Tumor Biol* 2015, **36**:1661–1665.

31. Yang M, Zhai X, Xia B, Wang Y, Lou G: **Long noncoding RNA CCHE1 promotes cervical cancer cell proliferation via upregulating PCNA**. *Tumor Biol* 2015.

32. Shihong Cao WLFLWZCQ: **Decreased expression of lncRNA GAS5 predicts a poor prognosis in cervical cancer**. *International Journal of Clinical and Experimental Pathology* 2014, **7**:6776.

33. Peng W, Gao W, Feng J: **Long noncoding RNA HULC is a novel biomarker of poor prognosis in patients with pancreatic cancer**. *Med Oncol* 2014, **31**:346.

34. Kim K, Jutooru I, Chadalapaka G, Johnson G, Frank J, Burghardt R, Kim S, Safe S: **HOTAIR is a negative prognostic factor and exhibits pro-oncogenic activity in pancreatic cancer.** *Oncogene* 2013, **32**:1616–1625.

35. Ma C, Nong K, Zhu H, Wang W, Huang X, Yuan Z, Ai K: **H19 promotes pancreatic cancer metastasis by derepressing let-7's suppression on its target HMGA2-mediated EMT.** *Tumor Biol* 2014, **35**:9163–9169.

36. Li R, Zhang L, Jia L, Duan Y, Li Y, Bao L, Sha N: **Long Non-Coding RNA BANCR Promotes Proliferation in Malignant Melanoma by Regulating MAPK Pathway Activation**. *PLoS ONE* 2014, **9**:e100893.

37. Lessard L, Liu M, Marzese DM, Wang H, Chong K, Kawas N, Donovan NC, Kiyohara E, Hsu S, Nelson N, Izraely S, Sagi-Assif O, Witz IP, Ma X-J, Luo Y, Hoon DSB: **The CASC15 Long Intergenic Noncoding RNA Locus Is Involved in Melanoma Progression and Phenotype Switching**. *Journal of Investigative Dermatology* 2015, **135**:2464–2474.

38. Mazar J, Zhao W, Khalil AM, Lee B, Shelley J, Govindarajan SS, Yamamoto F, Ratnam M, Aftab MN, Collins S, Finck BN, Han X, Mattick JS, Dinger ME, Perera RJ: **The Functional Characterization of Long Noncoding RNA SPRY4-IT1 in Human Melanoma Cells**. *Oncotarget* 2014, **5**:8959–8969.

39. Yuan SX, Yang F, Yang Y, Tao QF, Zhang J, Huang G, Yang Y, Wang RY, Yang S, Huo XS, Zhang L, Wang F, Sun SH, Zhou WP: **Long noncoding RNA associated with microvascular invasion in hepatocellular carcinoma promotes angiogenesis and serves as a predictor for hepatocellular carcinoma patients' poor recurrence-free survival after hepatectomy.** *Hepatology* 2012, **56**:2231–2241.

40. Xie H, Ma H, Zhou D: **Plasma HULC as a Promising Novel Biomarker for the Detection of Hepatocellular Carcinoma**. *BioMed Research International* 2013, **2013**:1–5.

41. Zhang T, Cao C, Wu D, Liu L: **SNHG3 correlates with malignant status and poor prognosis in hepatocellular carcinoma.** *Tumor Biol* 2015.

42. Huang M-D, Chen W-M, Qi F-Z, Xia R, Sun M, Xu T-P, Yin L, Zhang E-B, De W, Shu Y-Q: **Long non-coding RNA ANRIL is upregulated in hepatocellular carcinoma and regulates cell apoptosis by epigenetic silencing of KLF2.** *J Hematol Oncol* 2015, **8**:50.

43. Askarian-Amiri ME, Crawford J, French JD, Smart CE, Smith MA, Clark MB, Ru K, Mercer TR, Thompson ER, Lakhani SR, Vargas AC, Campbell IG, Brown MA, Dinger ME, Mattick JS: **SNORD-host RNA Zfas1 is a regulator of mammary development and a potential marker for breast cancer**. *RNA* 2011, **17**:878–891.

44. Silva JM, Boczek NJ, Berres MW, Ma X, Smith DI: **LSINCT5 is over expressed in breast and ovarian cancer and affects cellular proliferation.** *rnabiology* 2011, **8**:496–505.

45. Berteaux N, Aptel N, Cathala G, Genton C, Coll J, Daccache A, Spruyt N, Hondermarck H, Dugimont T, Curgy JJ, Forne T, Adriaenssens E: **A Novel H19 Antisense RNA Overexpressed in Breast Cancer Contributes to Paternal IGF2 Expression**. *Molecular and Cellular Biology* 2008, **28**:6731–6745.

46. Li H, Zhu L, Xu L, Qin K, Liu C, Yu Y, Su D, Wu K, Sheng Y: **Long noncoding RNA linc00617 exhibits oncogenic activity in breast cancer**. *Mol Carcinog* 2015:n/a–n/a.

47. Xu N, Chen F, Wang F, Lu X, Wang X, Lv M, Lu C: **Clinical significance of high expression of circulating serum lncRNA RP11-445H22.4 in breast cancer patients: a Chinese population-based study**. *Tumor Biol* 2015:1–7.

48. Tang H, Wu Z, Zhang J, Su B: **Salivary lncRNA as a potential marker for oral squamous cell carcinoma diagnosis.** *Mol Med Rep* 2013, **7**:761–766.

49. Fang Z, Wu L, Wang L, Yang Y, Meng Y, Yang H: **Increased expression of the long non-coding RNA UCA1 in tongue squamous cell carcinomas: a possible correlation with cancer metastasis**. *Oral Surgery, Oral Medicine, Oral Pathology and Oral Radiology* 2014, **117**:89–95.

50. Bo H, Gong Z, Zhang W, Li X, Zeng Y, Liao Q, Chen P, Shi L, Lian Y, Jing Y, Tang K, Li Z, Zhou Y, Zhou M, Xiang B, Li X, Yang J, Xiong W, Li G, Zeng Z: **Upregulated long non-coding RNA AFAP1-AS1 expression is associated with progression and poor prognosis of nasopharyngeal carcinoma**. *Oncotarget* 2015, **6**:20404–20418.
